# Research on Dynamic Characteristics of the RBBH System Based on Dynamics Model and Vibration Data Fusion

**DOI:** 10.3390/s22103806

**Published:** 2022-05-17

**Authors:** Shuilin Lin, Jianliang Sun, Chen Zhao, Yan Peng

**Affiliations:** National Cold Rolling Strip Equipment and Process Engineering Technology Research Center, Yanshan University, Qinhuangdao 066104, China; lslin@stumail.ysu.edu.cn (S.L.); zhaochen136@stumail.ysu.edu.cn (C.Z.); pengyan@ysu.edu.cn (Y.P.)

**Keywords:** strip mill, RBBH system, nonlinear dynamic model, dynamic response, vibration data

## Abstract

The roll-bearing-bearing housing (RBBH) system is one of the most common kernel structures used to determine strip mill stability and product surface quality in modern metallurgical machinery. To better understand dynamic characteristics of the RBBH system, this paper provides a nonlinear dynamic model and designs an engineering test platform on the RBBH system in the whole rolling process. First, a nonlinear dynamic model of the RBBH system supported by four-row rolling bearings under high speed and heavy load is established. Then, the method of combining Riccati transfer matrix and Newmark-*β* numerical integration is employed to solve nonlinear dynamic equations. After that, the engineering test platform is designed and assembled to capture and analyze the vibration signals of weathering steel (SPA-H) with finished thicknesses of 1.6 and 3.2 mm. Finally, the dynamic characteristics of the RBBH system are studied with the method of the dynamic model and vibration data fusion. The results show that the SPA-H with a finished thickness of 1.6 mm is rolled, the RBBH system fluctuates violently in both horizontal and vertical directions, and numerical results are highly consistent with experimental results in acceleration response, velocity response, and displacement response. In addition, the dynamic performance parameters of the four-row rolling bearing will also fluctuate greatly. Finally, there is significant interest to gain the benefits for the RBBH system design and mill stable rolling purposes.

## 1. Introduction

The instability of the RBBH system is one of the main reasons for misaligned loads of four-row rolling bearing, wear of the rolls, and vibration of the rolling mill. Moreover, a “necessary” minute gap between the mill window and bearing housing has made the instability problem increasingly prominent. The aforementioned phenomenon affects the quality and precision of strip products, reduces the stability of the RBBH system, and increases the vibration amplitude of the strip mill as well as unpredictable accidents [1,2].

The dynamic model of the RBBH system has attracted more and more attention from academic researchers and industrial operators in recent years. There are some excellent papers among these publications, aiming to provide guidance for researchers to model and analyze according to their research requirements. For example, Cao et al. [3] reviewed and critically discussed the current progress of mechanical model development of rolling bearing rotor (RBR) systems and identified future trends for research. Yang et al. [4] established a dynamic model of a rotor-bearing-casing system with a nonlinear rolling element bearing and studied the performance of rolling element bearing faults. Rusnak et al. [5] studied the nonlinear dynamics models of roll from various angles and achieved outstanding results. Liu et al. [6] deduced the friction between the roller and the strip, the torsional vibration of the gear pair and the horizontal vibration of the roller, and the dynamical equation of a horizontal–torsional coupled main drive system of a rolling mill. The authors of [7,8] summarized the different dynamic characteristics of the rolls such as variability, time variation, nonlinearity, and multi-constraint in the whole rolling process. Liu et al. [9,10] proposed a coupled vibration model for a roll-rolled piece. Wang et al. [11] studied the interface dynamic characteristics of a high-speed rolling mill work interface based on an unsteady lubrication process. Xu et al. [12] explained the dynamic vibration characteristics of the rolling mill under different rolling parameters. The influence of piecewise nonlinear factors on the vibration of rolls has also attracted extensive attention in the engineering field, such as friction, gap, and constraints [13,14].

In order to gain the dynamic characteristics of the RBBH system, in addition to the roll dynamics models that are studied, the field of rotor-bearing dynamics also needs to be explored. The rotor-bearing dynamics model generally consists of six models, i.e., the lumped-parameter model, quasi-static model, quasi-dynamic model, dynamic model, finite element model, and failure mechanics models. For instance, Sinou et al. [15] dealt with the nonlinear dynamic response of a flexible rotor supported by ball bearings. Moazen et al. [16] presented an improved nonlinear dynamic model of the contact forces and vibration response generated in defective rolling element bearings. Wang et al. [17] developed the quasi-static analysis model in order to analyze the performance of angular contact ball bearings. Gao et al. [18] introduced a comprehensive model considering the kinematics of the bearing components, the Hertzian contact between the rolling elements and raceways, the interaction between the rolling elements and cages, the hydrodynamic lubrication, and the thermal effects to study and forecast the over-skidding and skidding mechanisms. Than et al. [19] presented a unified method to predict nonlinear thermal characteristics of a high-speed spindle bearing subjected to a preload based on a quasi-static model and finite different method. Yang et al. [20] presented a five degrees-of-freedom quasi-dynamic model to analyze the relationship between the cage clearance and heating characteristics. Bovet et al. [21] present a modeling approach for predicting the internal dynamic behaviors of ball bearings under high moment loads. Cao et al. [22] proposed a new dynamic modeling method for ball-bearing rotor systems based on a rigid body element (RBE). Tu et al. [23] proposed a dynamic model to investigate the vibration response of a cylindrical roller bearing considering skidding. Liu et al. [24] established a skidding dynamics model of a rolling bearing to reveal the skidding characteristics. Singh et al. [25] provided insights into the mechanical mechanism by which defect-related impulsive forces, and consequently, vibrations are generated in defective rolling element bearings. Adeniyi et al. [26] created a numerical simulation for the oil/air flow in the region of a previously investigated aero-engine bearing between the inner race and the cage. 

The aforementioned papers have provided interesting reviews related to the dynamic behavior analysis of roll and the dynamic models of rotor-bearings. However, they have the following limitations: (1) The flexible support of bearings was rarely considered. (2) The RBBH system damping was greatly reduced due to bearings all being treated as rigid supports. (3) The dynamic characteristics of bearings were generally ignored by the existing literature, which in turn affected the variability of roll gaps and the dynamic response of mills. This paper fills these gaps and provides a systematic description of the dynamic characteristics of the RBBH system based on a dynamics model and vibration data fusion.

The remainder of this paper is organized as follows. Section 2 establishes the dynamic models of the RBBH system. Section 3 addresses the numerical calculation results and discussion. Details of the engineering test platform and results of the experiment are discussed in Section 4. Conclusions are drawn in Section 5.

## 2. Nonlinear Dynamic Models of the RBBH System

### 2.1. Engineering Background

With the development of high-end strip products such as weathering steel and automobile plates, the rolling force and rolling speed are gradually increasing. The production process of high speed and heavy load will inevitably induce drastic changes in the dynamic characteristics of the rolling equipment, the RBBH system is one of the typical representatives. The mutation of the dynamic characteristics reduces the service life of the rolling equipment and the surface quality of the product, which hinders the upgrading of the product. Revealing the dynamic characteristics of the RBBH system has become a major technical problem that the steel industry urgently needs to overcome. This paper describes and analyzes the dynamic characteristics of the hot strip mill as an example.

**Figure 1:**
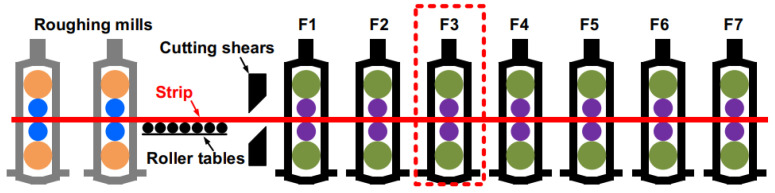
Diagram of equipment composition for hot rolling mill.

A hot strip mill is shown in Figure 1, including two four-high roughing mills, heat-preserving roller tables, cutting shears, seven four-high finishing mills (F1–F7), etc. The F3 finishing mill is selected as the motion analysis unit in this paper (red dashed box). The dynamic model of the RBBH system is established and the interaction between support rolls, rolling parts, main transmission system, mill housings, work rolls, and the flexible support of the bearing are considered.

**Figure 2:**
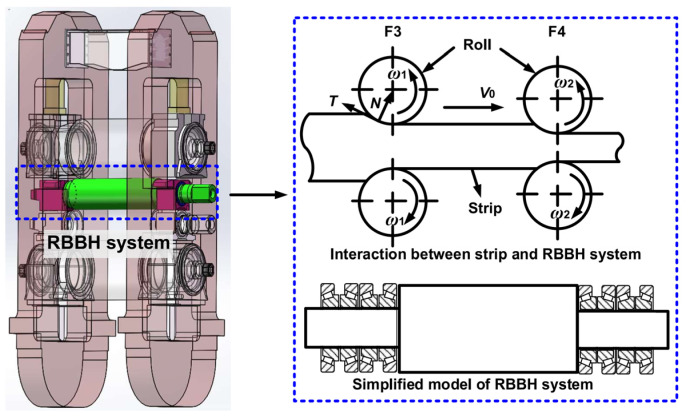
Analysis model of the RBBH system.

Strip rolling must undergo three production processes, i.e., the biting steel process, stable process, and polishing steel. In the whole rolling process, rolling speed and rolling force change rapidly with the running of the strip, resulting in variability in the dynamic stiffness and damping of support bearings; at the same time, the dynamic parameters of support bearings will cause the transient response of rolling. In order to express the relationship between the parts in the RBBH system intuitively, the analysis model is established (Figure 2). The RBBH system consists of the rolls, bearings, and housings, where the bearings and housings ensure the operational accuracy of the rolls and are an important influence on the surface quality of the product. In the same way, the operating condition of the rolls also affects the accuracy and life of the bearings and housings. The components in the RBBH system affect each other as a whole. The roll and the four-row bearings are regarded as an elastic structure and the flexible support, respectively. The following assumptions are made for the convenience of the solution.

(1) The roll is an isotropic beam of equal cross-section; the longitudinal displacement along the axis of the roll and the elastic deformation of the roll along the rolling line caused by the horizontal load are ignored.
(2) The influence of roll moment of inertia and shear deformation are ignored.
(3) The contact between the work roll and the support roll is elastic contact.

### 2.2. The RBBH System Modeling

**Figure 3:**
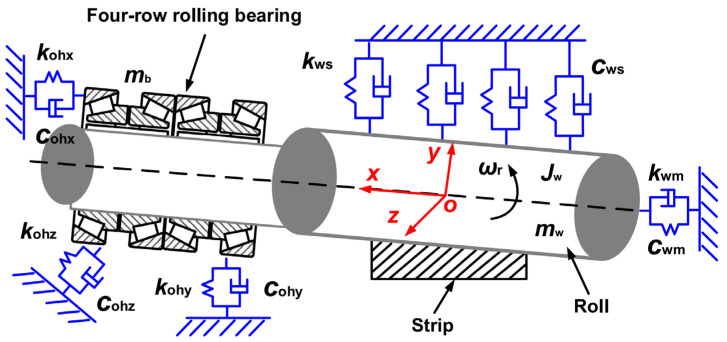
Nonlinear dynamic model of the RBBH system.

Incorporating the flexible support of four-row rolling element bearings, the dynamic model of the RBBH system is established as shown in Figure 3. *m*_w_ and *m*_b_ represent the equivalent inertial masses of the work roll and the four-row bearing and bearing housing. *J*_w_ and *ω*_r_ are the equivalent moment of inertia and rolling angular velocity of the work roll, respectively. *k*_ohx_, *k*_ohy_, *k*_ohz_, *c*_ohx_, *c*_ohy_, and *c*_ohz_ denote the equivalent stiffness and equivalent damping between the outer ring of the bearing and the bearing housing, respectively. *k*_ws_ and *c*_ws_ are the equivalent stiffness and equivalent damping between the work roll and the support roll, and *k*_wm_ and *c*_wm_ are the equivalent stiffness and equivalent damping of the connection between the work roll and the main drive system, respectively.

The dynamic characteristics of the four-row bearings and the roll in the RBBH system affect each other, and they provide an operating environment for each other. In order to obtain a more versatile RBBH system dynamic model and considering the contact stiffness and damping of the bearing, a universal four-row bearing dynamic model is established and the following assumptions are made. (1) The geometric center of each part coincides with the center of mass. (2) The inner ring and the roll have an interference fit. (3) The roll rotates around the center of mass and revolves around the axis of the inner ring. The center of mass of the roll and the inner ring can move in three-dimensional translation. The cage rotates around its central axis. The angular velocity is a fixed value and the outer ring is fixed. (4) The axial force is ignored. (5) The lubricating oil’s viscosity, viscosity–temperature, viscosity–pressure coefficient, density, and other parameters are confirmed. (6) The roller sliding in the bearing is not considered. (7) There is no radial clearance.

**Figure 4:**
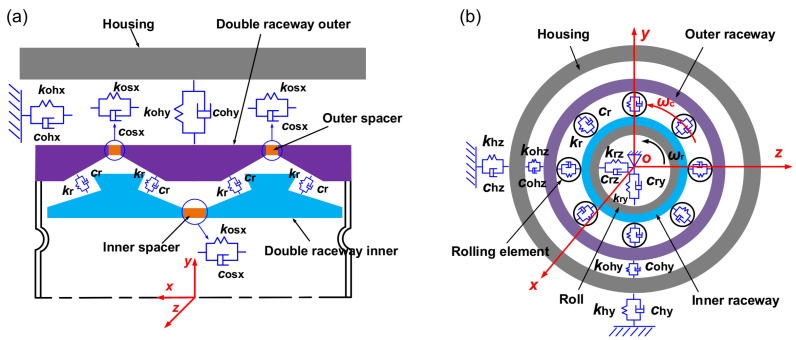
Dynamic model: (**a**) rolling direction and (**b**) axial direction.

The variable flexibility vibration of the rolling bearing has a great impact on the RBBH system, and it is necessary to model the bearing separately. The front view and side view of the four-row bearing dynamic model are shown in Figure 4. *k*_r_ and *c*_r_ are the equivalent contact stiffness and damping of the rolling element and the ferrule, respectively. *k*_osx_ and *c*_osx_ are the equivalent contact stiffness and damping of the inner and outer spacers, respectively. *k*_ohz_, *c*_ohz_, *k*_ohy_, and *c*_ohy_ are the equivalent stiffness and damping of the outer ring and the bearing housing in the *z* and *y* directions, respectively. *k*_rz_ and *c*_rz_ are the equivalent stiffness and damping of the roll, respectively. *k*_hz_, *c*_hz_, *k*_hy_, and *c*_hy_ are the equivalent stiffness and damping of the bearing and mill rack in the *z* and *y* directions, respectively. *ω*_r_ is the rolling angular velocity of the roll and *ω*_c_ is the angular velocity of the roller revolution.

The dynamic control equation is established using the RBBH system dynamic model as

(1) $$M\ddot{S}\left(t\right)+C\dot{S}\left(t\right)+KS\left(t\right)+P_{b}=Q_{w}$$

where *M* is the RBBH system mass matrix, *C* is the damping matrix, *K* is the stiffness matrix, *S*(*t*) is the displacement matrix, *P*_b_ is the external load matrix of the four-row bearing, and *Q*_w_ is the external load matrix of the roll. The following will expand the description of each item in Equation (1). The displacement matrix of the RBBH system is expressed as

(2) $$S\left(t\right)={\left[y_{r}\left(t\right)z_{r}\left(t\right)y_{i}\left(t\right)z_{i}\left(t\right)y_{o}\left(t\right)z_{o}\left(t\right)y_{h}\left(t\right)z_{h}\left(t\right)\right]}^{T}$$

where *y*(*t*) and *z*(*t*) are the displacement vectors of the components of the system in the *y* and *z* directions (that is, the vertical direction and the rolling direction) over time. The quality matrix of the system is

(3) $$M=\left[\begin{array}{l} M_{w}\;0\;\;\;0\\ \;\;0\;M_{b}\;0\\ \;\;0\;\;0\;M_{h} \end{array}\right]$$

(4) $$\left\{\begin{array}{l} M_{w}=\left[\begin{array}{l} m_{w}+m_{i}\;\;\;\;0\\ \;\;\;\;0\;\;m_{w}+m_{i} \end{array}\right]\\ M_{b}=\left[\begin{array}{l} m_{o}+m_{r}\;\;\;\;0\\ \;\;\;0\;\;m_{o}+m_{r} \end{array}\right]\\ M_{h}=\left[\begin{array}{l} m_{h}\;\;\;\;0\\ \;\;\;\;0\;m_{h} \end{array}\right] \end{array}\right.$$

Here, *m*_w_, *m*_i_, *m*_o_, *m*_r_, and *m*_h_ are the equivalent masses of the roll, inner ring, outer ring, rolling element, and bearing housing, respectively. Due to the interference fit between the inner ring and the roll, the equivalent mass of the inner ring is merged into the roll mass matrix. 

The system damping matrix is expressed as

(5) $$C=\left[\begin{array}{l} C_{w}\;\;\;-C_{b}\;\;\;0\\ -C_{b}\;C_{b}+C_{\mathrm{oh}}\;\;-C_{\mathrm{oh}}\\ 0\;\;\;-C_{\mathrm{oh}}\;\;\;C_{\mathrm{oh}}+C_{h} \end{array}\right]$$

(6) $$\left\{\begin{array}{l} C_{w}=\left[\begin{array}{l} c_{w}+c_{i}\;\;\;\;0\\ \;\;\;\;0\;\;c_{w}+c_{i} \end{array}\right]\text{,}C_{b}=\left[\begin{array}{l} c_{o}+c_{r}\;\;\;\;0\\ \;\;\;\;0\;\;c_{o}+c_{r} \end{array}\right]\\ C_{\mathrm{oh}}=\left[\begin{array}{l} c_{\mathrm{oh}}\;\;\;0\\ \;0\;c_{\mathrm{oh}} \end{array}\right]\text{,}C_{h}=\left[\begin{array}{l} c_{h}\;0\\ \;0\;c_{h} \end{array}\right] \end{array}\right.$$

Here, *c*_w_, *c*_i_, *c*_o_, *c*_r_, *c*_oh_, and *c*_h_ are the equivalent damping of the roller, bearing inner ring, outer ring, rolling element, outer ring and bearing housing, and bearing housing, respectively.

The system stiffness matrix is

(7) $$K=\left[\begin{array}{l} K_{w}\;\;\;0\;\;\;\;0\\ 0\;\;K_{r}+K_{\mathrm{oh}}\;\;-K_{\mathrm{oh}}\\ 0\;\;\;-K_{\mathrm{oh}}\;\;\;\;K_{h}+K_{\mathrm{oh}} \end{array}\right]$$

(8) $$\left\{\begin{array}{l} K_{w}=\left[\begin{array}{l} k_{w}+k_{i}\;\;\;\;0\\ \;\;\;\;0\;\;k_{w}+k_{i} \end{array}\right]\text{,}K_{r}=\left[\begin{array}{l} k_{r}\;\;\;\;0\\ \;0\;\;k_{r} \end{array}\right]\\ K_{\mathrm{oh}}=\left[\begin{array}{l} k_{\mathrm{oh}}\;\;\;\;0\\ \;0\;\;k_{\mathrm{oh}} \end{array}\right]\text{,}K_{h}=\left[\begin{array}{l} k_{h}\;\;\;\;0\\ \;0\;\;k_{h} \end{array}\right] \end{array}\right.$$

Here, *k*_w_, *k*_i_, *k*_r_, *k*_oh_, and *k*_h_ are the equivalent stiffness of the roll, inner ring, outer ring, rolling element, outer ring and bearing housing, and bearing housing, respectively. The external load matrix of bearings and roll is:

(9) $$P_{b}=\left[\begin{array}{l} P_{\mathrm{bi}}\;0\\ \;0\;P_{\mathrm{bo}} \end{array}\right],\;Q_{w}=\left[\begin{array}{l} Q_{\mathrm{ws}}\;0\\ \;0\;Q_{\mathrm{wb}} \end{array}\right]$$

(10) $$\left\{\begin{array}{l} P_{\mathrm{bi}}=\left[\begin{array}{l} p_{\mathrm{bi}}\;0\\ \;0\;p_{\mathrm{bo}} \end{array}\right]\text{,}P_{\mathrm{bo}}=\left[\begin{array}{l} -p_{\mathrm{bi}}\;0\\ \;0\;\;\;-p_{\mathrm{bo}} \end{array}\right]\\ Q_{\mathrm{ws}}=\left[\begin{array}{l} F_{\mathrm{sy}}\;0\\ \;0\;F_{\mathrm{sz}} \end{array}\right]\text{,}Q_{\mathrm{wb}}=\left[\begin{array}{l} F_{\mathrm{by}}\;0\\ \;0\;F_{\mathrm{bz}} \end{array}\right] \end{array}\right.$$

Here, *p*_bi_, *p*_bo_, *F*_sy_, *F*_sz_, *F*_by_, and *F*_bz_ are the external loads of the bearing inner ring, the bearing outer ring, the strip acting on the roll, and support roll on the work roll, respectively.

### 2.3. Methodology

**Figure 5:**
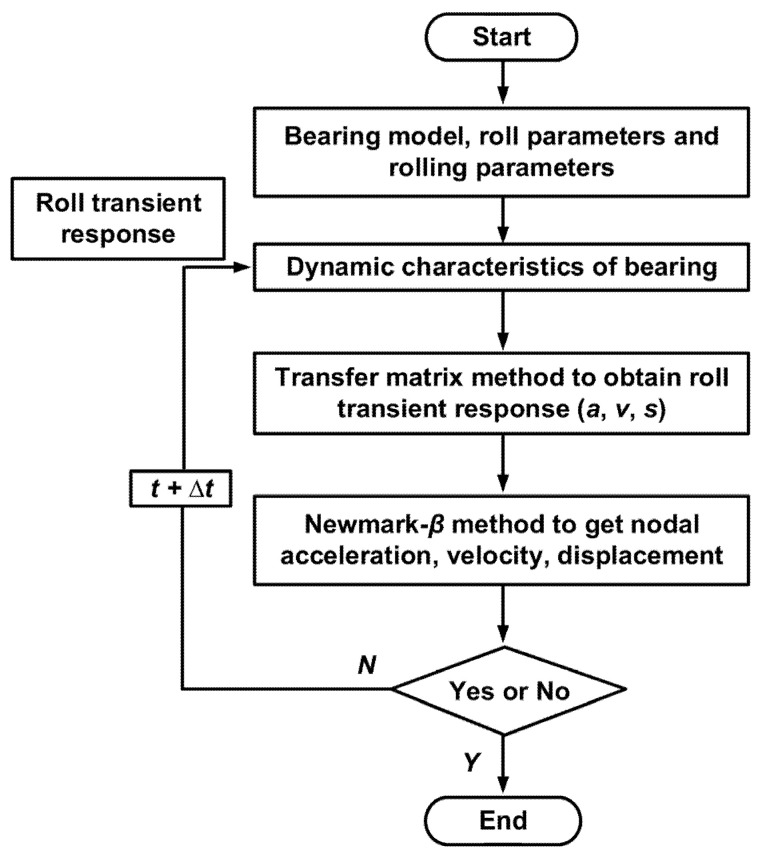
The overall calculation flow chart of the transient response.

The dynamic response of the RBBH system will change with the rolling speed, rolling force, and other working conditions, which will affect the dynamic characteristics of support bearings. In order to accurately calculate the dynamic response of the system, the rolling speed, rolling force, and other working conditions are used as the initial conditions for solving the dynamic equations. Then, it is substituted into the transient response equation of the RBBH system, and the calculation is repeated until the system response is stable. The flowchart is shown in Figure 5.

The Riccati transfer matrix method is combined with the Newmark-*β* numerical integration method. According to the Newmark-*β* method, *γ* = 0.5, and the specific formula is

(11) $${\ddot{S}}_{t+\Delta t}=\frac{1}{\beta^{\prime }\Delta t^{2}}\left\{\left.S_{t+\Delta t}-S_{t}\right\}\right.-\frac{1}{\beta^{\prime }\Delta t}{\dot{S}}_{t}-\left(\frac{1}{2\beta^{\prime }}-1\right){\ddot{S}}_{t}$$

(12) $${\dot{S}}_{t+\Delta t}={\dot{S}}_{t}+\frac{1}{2}\Delta t\left({\ddot{S}}_{t}+{\ddot{S}}_{t+\Delta t}\right)$$

where *β’* is the parameter of the Newmark-*β* method and *S* is the generalized coordinate of the RBBH system. At an arbitrary rolling instant *t*, the displacement $S_{t}$, speed ${\dot{S}}_{t}$, and acceleration ${\ddot{S}}_{t}$ of the system are known in the numerical solution. The instantaneous rolling displacement $S_{t+\Delta t}$, the speed ${\dot{S}}_{t+\Delta t}$, and acceleration ${\ddot{S}}_{t+\Delta t}$ of t+Δt can be obtained from Equations (11) and (12).

At instant *S*_0_, the initial condition of motion is the acceleration of each node and each supporting mass, by which the velocity and displacement can be calculated. The initial acceleration formula is

(13) $$\left\{\begin{array}{l} \ddot{x}\\ \ddot{y}\\ \ddot{z} \end{array}\right\}=\frac{1}{m_{i}}\left[\begin{array}{l} -K_{j}\left\{\begin{array}{l} x_{i}-x_{hj}\\ y_{i}-y_{hj}\\ z_{i}-z_{hj} \end{array}\right\}-C_{j}\left\{\begin{array}{l} {\dot{x}}_{i}-{\dot{x}}_{hj}\\ {\dot{y}}_{i}-{\ddot{y}}_{hj}\\ {\ddot{z}}_{i}-{\ddot{z}}_{hj} \end{array}\right\}+{\left(k_{1}\right)}_{i-1}{\left\{\begin{array}{l} x\\ y\\ z \end{array}\right\}}_{i-1}+{\left(k_{2}\right)}_{i-1}{\left\{\begin{array}{l} \alpha \\ \beta \\ \gamma \end{array}\right\}}_{i-1}-\\ \left[{\left(k_{1}\right)}_{i-1}+{\left(k_{1}\right)}_{i}\right]{\left\{\begin{array}{l} x\\ y\\ z \end{array}\right\}}_{i}+\left[{\left(k_{2}\right)}_{i-1}+{\left(k_{2}\right)}_{i}\right]{\left\{\begin{array}{l} \alpha \\ \beta \\ \gamma \end{array}\right\}}_{i}+\\ {\left(k_{1}\right)}_{i}{\left\{\begin{array}{l} x\\ y\\ z \end{array}\right\}}_{i+1}-{\left(k_{2}\right)}_{i}{\left\{\begin{array}{l} \alpha \\ \beta \\ \gamma \end{array}\right\}}_{i+1}+{\left\{\begin{array}{l} F_{x}\left(t_{0}\right)\\ F_{y}\left(t_{0}\right)\\ F_{z}\left(t_{0}\right) \end{array}\right\}}_{i} \end{array}\right]$$

At the instant *t* of the RBBH system dynamics model, if the calculation node has no support, all items related to the support are zero. The differential equation of motion of a node j can be expressed as

(14) $$K_{j}\left\{\begin{array}{l} x_{j}\\ y_{j}\\ z_{j} \end{array}\right\}+C_{j}\left\{\begin{array}{l} {\dot{x}}_{j}\\ {\dot{y}}_{j}\\ {\dot{z}}_{j} \end{array}\right\}+m_{j}\left\{\begin{array}{l} \ddot{x}\\ \ddot{y}\\ \ddot{z} \end{array}\right\}+\left\{\begin{array}{l} P_{bx}(t)\\ P_{by}(t)\\ P_{bz}(t) \end{array}\right\}={\left\{\begin{array}{l} Q_{wx}(t)\\ Q_{wy}(t)\\ Q_{wz}(t) \end{array}\right\}}_{j}$$

## 3. Numerical Results and Discussion

### 3.1. Model Parameters

The F3 finishing mill of a hot rolling mill is analyzed as an application example, and the flexible support bearing model of the RBBH system is M255449DW/10/10D. Table 1 and Table 2 give the structural parameters and the material parameters of each component of the RBBH system, respectively.

### 3.2. Rolling Process Parameters

SPA-H is widely used in important infrastructure such as railways, vehicles, and bridges, and is one of the most important products in steel production. Therefore, we chose SPA-H as the experimental product. Second, the RBBH system exhibits different dynamic responses when different finished thicknesses of SPA-H are rolled, and the rolling conditions are different. In order to study the effect of different influencing factors (e.g., rolling force, rolling speed) on the dynamic characteristics of the RBBH system, we chose the finished thicknesses of 1.6 and 3.2 mm SPA-H as the experimental conditions, respectively, because the system showed rich dynamic characteristics when the 1.6 mm was rolled, and on the contrary, the 3.2 mm was more stable overall. Therefore, these two finished thicknesses were chosen as the experimental conditions for the study. In addition to the analysis of the SPA-H of 1.6 and 3.2 mm in this section, the influence of rolling force, rolling speed, and product thickness on the dynamic characteristics of the RBBH system are also considered. 

**Figure 6:**
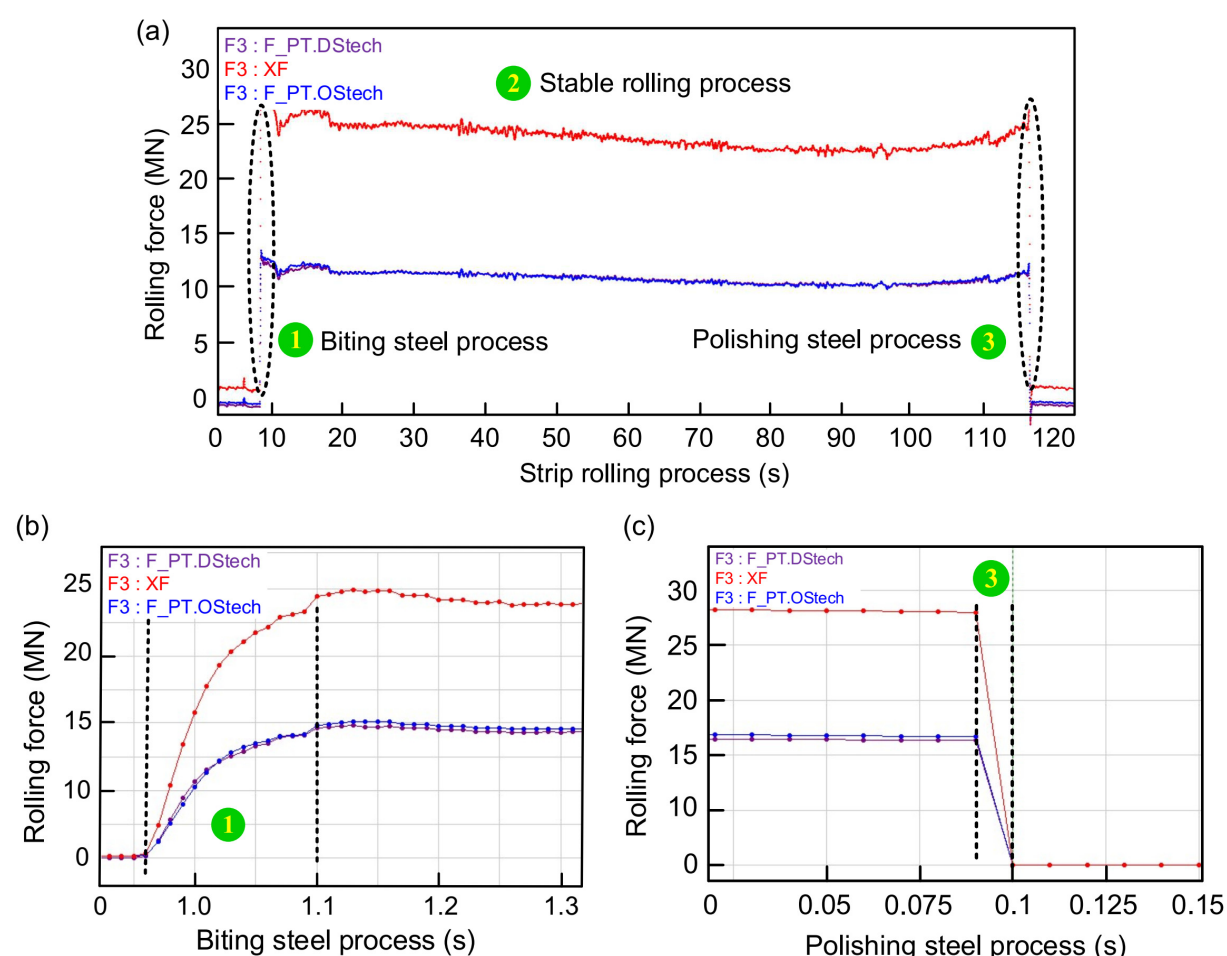
The change in rolling force when the finished product is 1.6 mm: (**a**) total rolling force, (**b**) biting rolling force, and (**c**) polishing rolling force.

**Figure 7:**
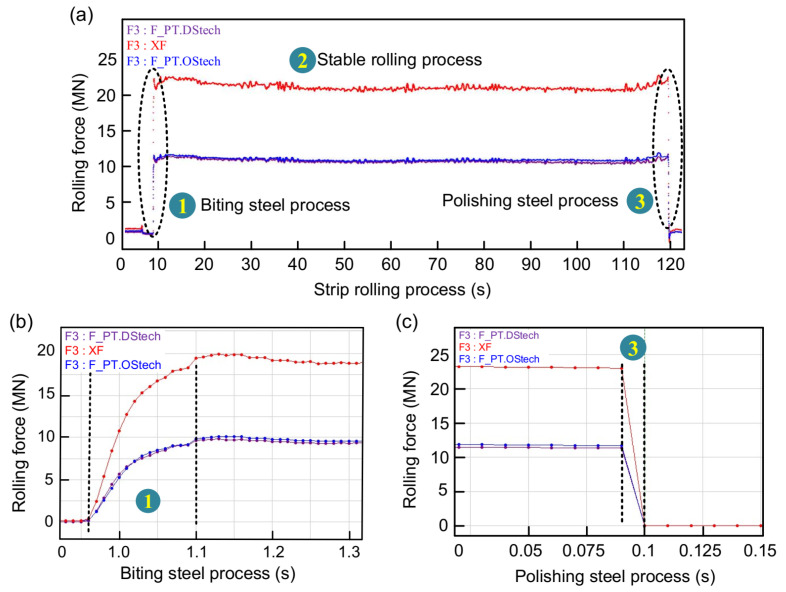
The change in rolling force when the finished product is 3.2 mm: (**a**) total rolling force, (**b**) biting rolling force, and (**c**) polishing rolling force.

When the SPA-H of 1.6 and 3.2 mm are rolled, the changes in rolling force are given in Figure 6 and Figure 7. It can be seen from Figure 6a that the stable process rolling force fluctuation is very small and can be ignored. Figure 6b shows that the biting time lasts for 0.25 s and the total rolling force soars from 0 to 25 MN. Figure 6c shows that the polishing steel time lasts only 0.025 s and the rolling force is instantly reduced from 30 to 0 MN. Figure 7 presents the rolling force distribution with a finished product thickness of 3.2 mm. Compared with the rolling force with a finished product thickness of 1.6 mm, the value is reduced by approximately 5 MN and the rolling process is more stable. 

**Figure 8:**
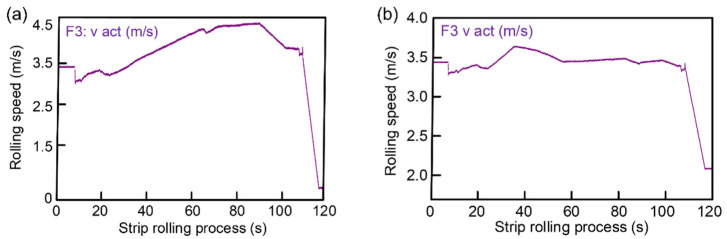
The change in rolling speed: (**a**) 1.6 mm and (**b**) 3.2 mm.

As shown in Figure 8a, the rolling speed of 1.6 mm continues to increase and the maximum is close to 4.5 m/s. Figure 8b shows that the rolling speed of 3.2 mm is maintained at about 3.5 m/s. Compared with 1.6 mm, the rolling process is relatively stable.

### 3.3. Numerical Results and Discussion

**Figure 9:**
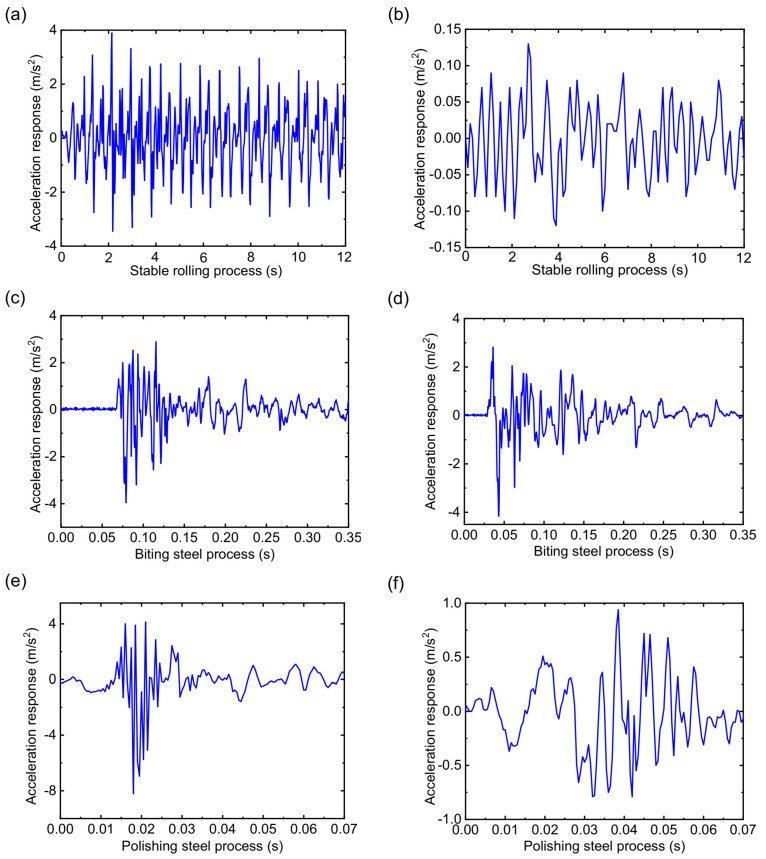
Transient acceleration response of the system: (**a**) stable rolling process of the 1.6 mm, (**b**) stable rolling process of the 3.2 mm, (**c**) biting steel process of the 1.6 mm, (**d**) biting steel process of the 3.2 mm, (**e**) polishing steel process of the 1.6 mm, and (**f**) polishing steel process of the 3.2 mm.

This section discusses the dynamic characteristics of the RBBH system with the method of numerical analysis from three dynamic indicators, i.e., acceleration response, velocity response, and displacement response, in which the impact of dynamic stiffness and dynamic damping of the bearing are considered. Part of the rolling process is selected for analysis (12 s) due to the slight response fluctuation in the stable rolling process. The biting steel process and the polishing steel process are analyzed according to the actual process. Figure 9 shows the acceleration response when the SPA-H of 1.6 and 3.2 mm are rolled. Figure 9a,b show the stable rolling process. It can be seen from Figure 9b that the response amplitude is stable without sharp fluctuations and the maximum amplitude is less than 0.15 m/s^2^ in the process of the 3.2 mm being rolled. When the SPA-H of 1.6 mm is rolled, the response amplitude is nearly 20 times that of 3.2 mm, from which it can be inferred that the RBBH system is subject to severe periodic shocks. As shown in Figure 9c,d, the duration of the entire biting steel process is about 0.1 s. It can be seen that no matter what kind of steel is rolled, it will cause a sudden increase in acceleration response, which has the most severe transient impact on bearings and rolls. Figure 9e,f show the polishing steel process. The excessive rolling speed and rolling force cause the RBBH system to exhibit severe fluctuations in the entire polishing steel process (0.7 s).

**Figure 10:**
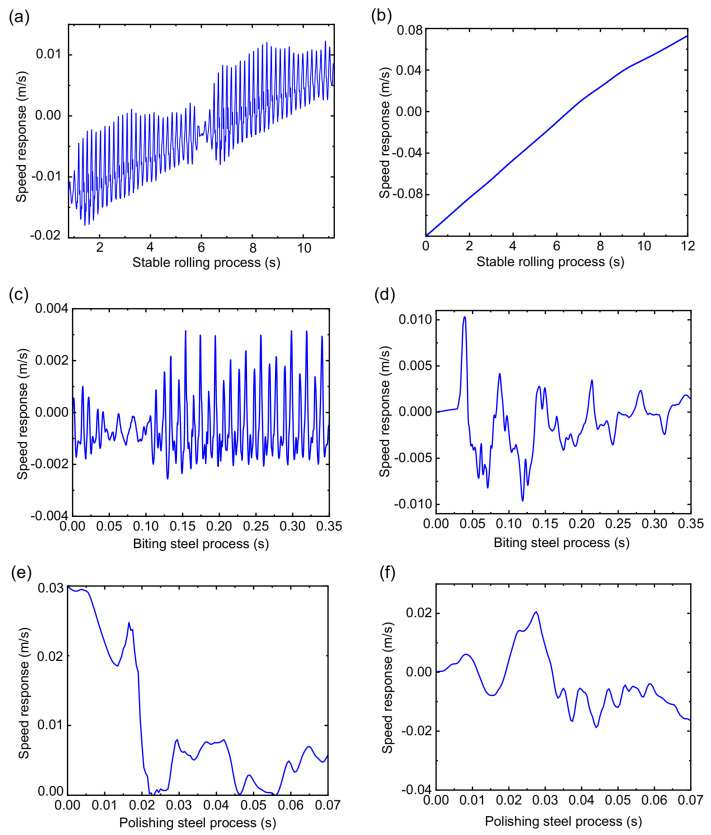
Transient speed response of the system: (**a**) stable rolling process of the 1.6 mm, (**b**) stable rolling process of the 3.2 mm, (**c**) biting steel process of the 1.6 mm, (**d**) biting steel process of the 3.2 mm, (**e**) polishing steel process of the 1.6 mm, and (**f**) polishing steel process of the 3.2 mm.

The speed response with the SPA-H of 1.6 and 3.2 mm is presented in Figure 10. The stability of the RBBH system decreases as the rolling speed continues to increase. The RBBH system has different speed characteristics when different strips are rolled. The speed change always fluctuates at the center line and forms a symmetrical trend due to the limitation of the gap between bearing housing and mill window. As shown in Figure 10a, the speed response of the RBBH system presents period changes, and is symmetrical at 0 m/s. On the contrary, the SPA-H of 3.2 mm shows a relatively stable state as shown in Figure 10b. During the biting and polishing steel process, the speed response presents complex changes as shown in Figure 10c–f because of many influencing factors. In addition, it can be known from the dynamic theory of gears and bearings that the increase in the rolling speed will lead to the decrease in the stability of the RBBH system. One of the applications of speed response is that reducing the rolling speed can well suppress the vibration of the rolling mill. 

**Figure 11:**
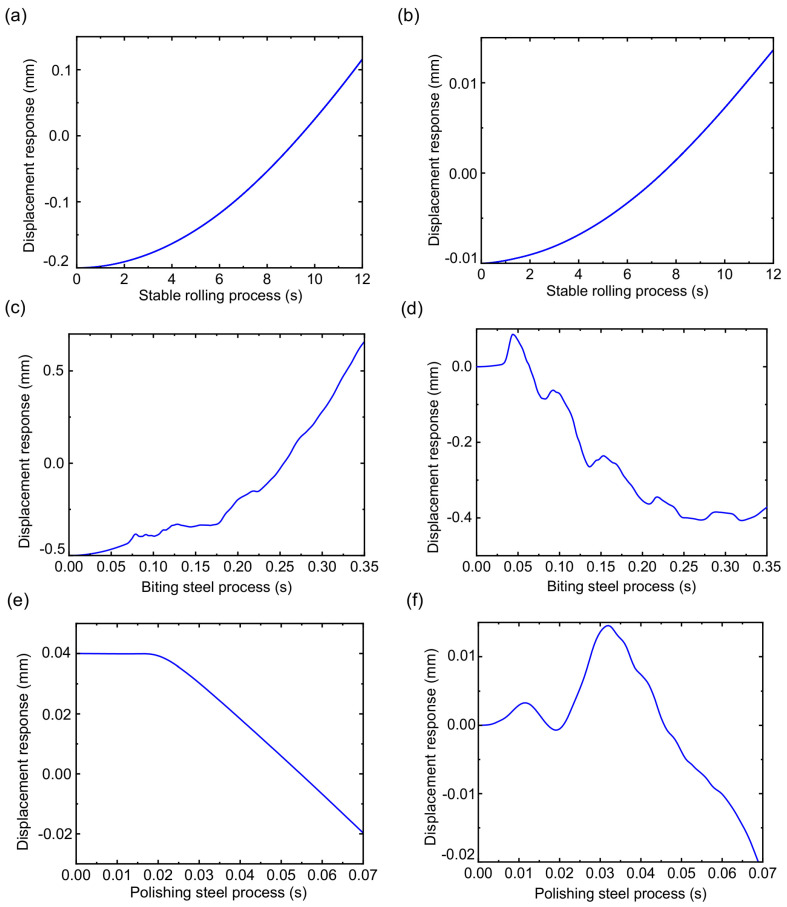
Transient displacement response of the system: (**a**) stable rolling process of the 1.6 mm, (**b**) stable rolling process of the 3.2 mm, (**c**) biting steel process of the 1.6 mm, (**d**) biting steel process of the 3.2 mm, (**e**) polishing steel process of the 1.6 mm, and (**f**) polishing steel process of the 3.2 mm.

The displacement response of the RBBH system is given in Figure 11. The RBBH system is not a simple rigid translation or rotation in the actual rolling process, but a wing form combining translation and rotation. Figure 11a,b show the displacement fluctuation in the RBBH system during stable rolling. It can be seen that the system only has an offset of about 0.02 mm in the direction of the roll axis because of the stable rolling force and rolling speed. As shown in Figure 11c,d, although the RBBH system completes a fluctuation cycle in the gap between the bearing housing and the stand window within 0.35 s of the biting process, the fluctuation range will not exceed the actual gap. In the polishing steel process, the RBBH system displacement undergoes a sudden change within 0.07 s as the strip leaves the roll gap and then returns to a stable value at low speeds waiting for the next strip to pass through, as shown in Figure 11e,f.

## 4. Experimental Validation

### 4.1. Model Parameters

**Figure 12:**
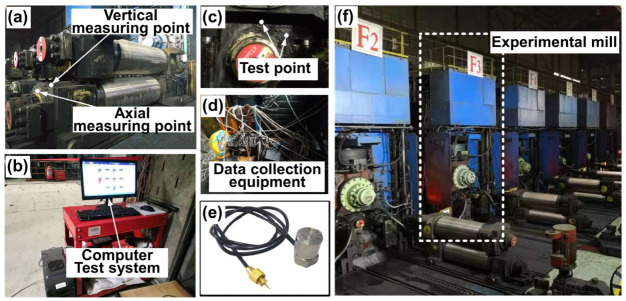
Engineering experiment test platform: (**a**) the RBBH system, (**b**) test instrument, (**c**) test point, (**d**) test system, (**e**) piezoelectric acceleration sensor, and (**f**) experimental rolling mill.

In order to verify the accuracy of numerical calculations an engineering test platform was designed and constructed to collect and analyze vibration data through piezoelectric acceleration sensors. The test platform is shown in Figure 12. Figure 12e presents the piezoelectric acceleration sensor, which is suitable for dynamic testing of the finishing mill with frequent roll changes because of high sensitivity and portability. The sensor converts the acceleration signal into a charge signal and transmits the dynamic signal to the computer (see Figure 12b) for recording and display through the charge amplifier and capture card. Accelerometers are installed on horizontal and vertical directions of the bearing housing on the operating side, as shown in Figure 12a,c. 

This test experiment covers the whole process of the rolling strip, including three typical production processes of steel biting, steel stable rolling, and steel polishing. This paper takes the finished product thicknesses of 1.6 and 3.2 mm as examples for analysis. The rolling mill exhibits violent vibration and the vibration feeling is strong on site when the 1.6 mm product is rolled. For comparative analysis, dynamic data of the 3.2 mm are also collected and analyzed.

### 4.2. Results and Discussion

The vibration signals of the RBBH system are collected by sensors installed in the horizontal and vertical directions and processed for signal denoising. The dynamic information in the axial direction is ignored because the locking device fixes the displacement of the RBBH system in the axial direction during the signal acquisition process. Then, the correctness and validity of the dynamic model are verified by three indicators of acceleration response, velocity response, and displacement response. H and V represent the horizontal and vertical directions, respectively.

**Figure 13:**
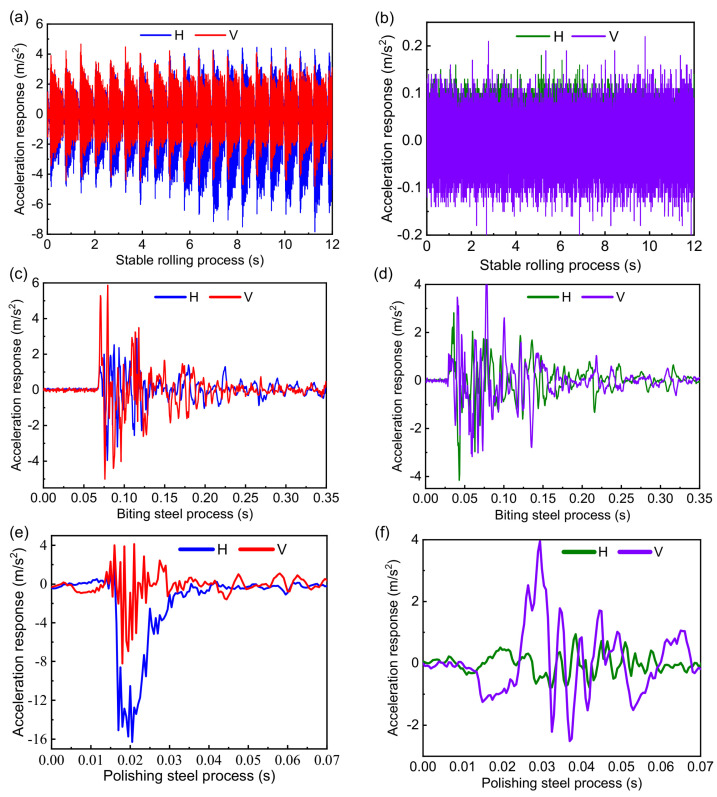
Transient acceleration response of the system when the finished weathering steel thicknesses are 1.6 and 3.2 mm: (**a**) stable rolling process of the 1.6 mm, (**b**) stable rolling process of the 3.2 mm, (**c**) biting steel process of the 1.6 mm, (**d**) biting steel process of the 3.2 mm, (**e**) polishing steel process of the 1.6 mm, and (**f**) polishing steel process of the 3.2 mm.

Signal processing results of the acceleration response in the biting process, the stable rolling process, and the polishing process are provided in Figure 13. As shown in Figure 13a,b, the stable rolling process shows obvious periodic fluctuations when the 1.6 mm is rolled, which leads to the changes in the dynamic characteristics and dynamic stiffness of the bearing. The acceleration response in the horizontal direction gradually increases to 4 m/s^2^ with the rolling time, and the essence is that the rolling speed gradually increases with the rolling process, which leads to a decrease in the stability of the system. The acceleration response in the vertical direction is always around 2 m/s^2^, essentially because of the limitation of the RBBH system by the support rollers in the vertical direction. The results of the experiment are less than half of the numerical results in both the horizontal and vertical directions, indicating that the numerical results are coupled results with the horizontal and vertical directions. Figure 13c,d show the acceleration response of the biting process. As can be seen from Figure 13c,d, the acceleration response of the biting process remains basically the same regardless of whether the finished thickness is 1.6 or 3.2 mm, which increases instantaneously to a peak and then gradually decreases to zero. Although the rolling process shows completely different dynamic characteristics under the premise that the strip thickness, rolling force, rolling speed, and tension are different, the overall pattern is the same. The rolling speed instantly drops from 3.5 to 0 m/s whether it is 1.6 or 3.2 mm in the polishing steel process, resulting in the dynamic characteristics of the RBBH system being basically the same, as seen in Figure 13e,f. However, the instantaneous amplitude of the polishing steel is much greater than 3.2 mm when the finished thickness of 1.6 mm is rolled, because the rolling conditions of 1.6 mm are much worse than 3.2 mm resulting in a more sensitive system stability. The experimental results are basically consistent with the numerical results in the acceleration response.

In summary, the acceleration response of the RBBH system is greatly affected by the rolling conditions, which in turn are closely related to the rolled product.

**Figure 14:**
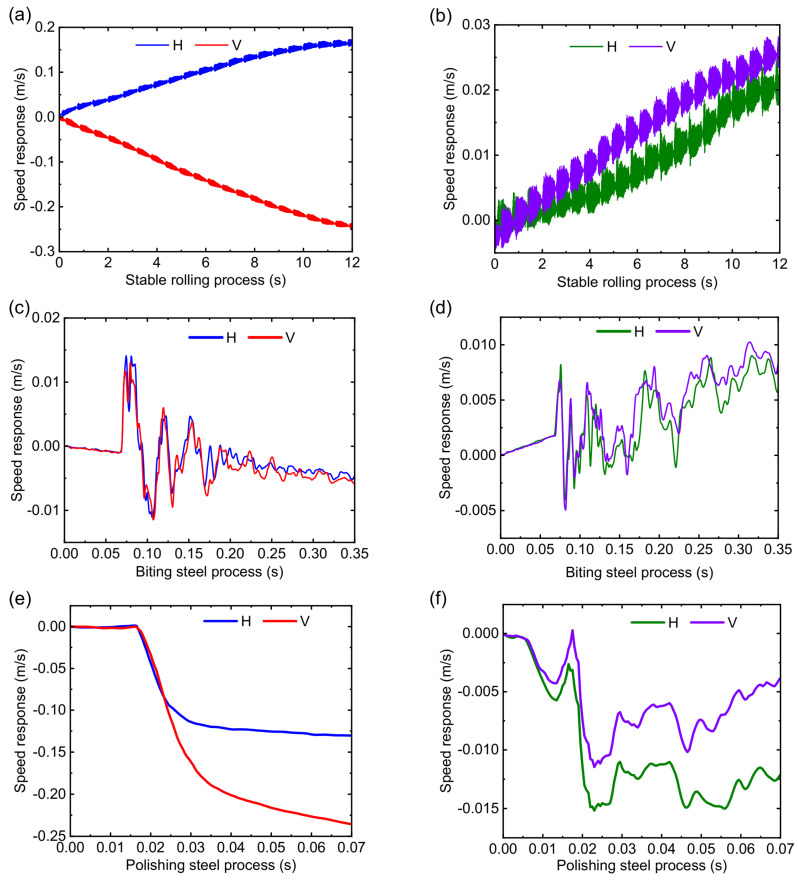
Transient speed response of the system when the finished weathering steel thickness is 1.6 and 3.2 mm: (**a**) stable rolling process of the 1.6 mm, (**b**) stable rolling process of the 3.2 mm, (**c**) biting steel process of the 1.6 mm, (**d**) biting steel process of the 3.2 mm, (**e**) polishing steel process of the 1.6 mm, and (**f**) polishing steel process of the 3.2 mm.

The speed response of the 1.6 and the 3.2 mm is presented in Figure 14. As shown in Figure 14a,b, horizontal and vertical speeds remain symmetrical when 1.6 mm is in the stable rolling process, while 3.2 mm has positive fluctuations in both horizontal and vertical speeds. The speed fluctuation range of 0.02 m/s for a finished product thickness of 3.2 mm is normal, but the speed fluctuation of 1.6 mm is significant, which mainly originates from the change of rolling force and rolling speed. Figure 14c,d shows the speed response of the biting process. Whether it is 1.6 or 3.2 mm, the fluctuation directions of the horizontal speed and the vertical speed are basically the same, which suddenly increase from 0 m/s to the maximum value, and then gradually and regularly drop to a stable value. Figure 14e,f show the speed response of the polishing process. It can be seen from the figure that both the horizontal speed and the vertical speed start to fluctuate from a value of 0, and the fluctuations are irregular, which may be related to the poor control of the tail of the rolled product. Both the horizontal and vertical speed responses are consistent with the numerical calculations. 

**Figure 15:**
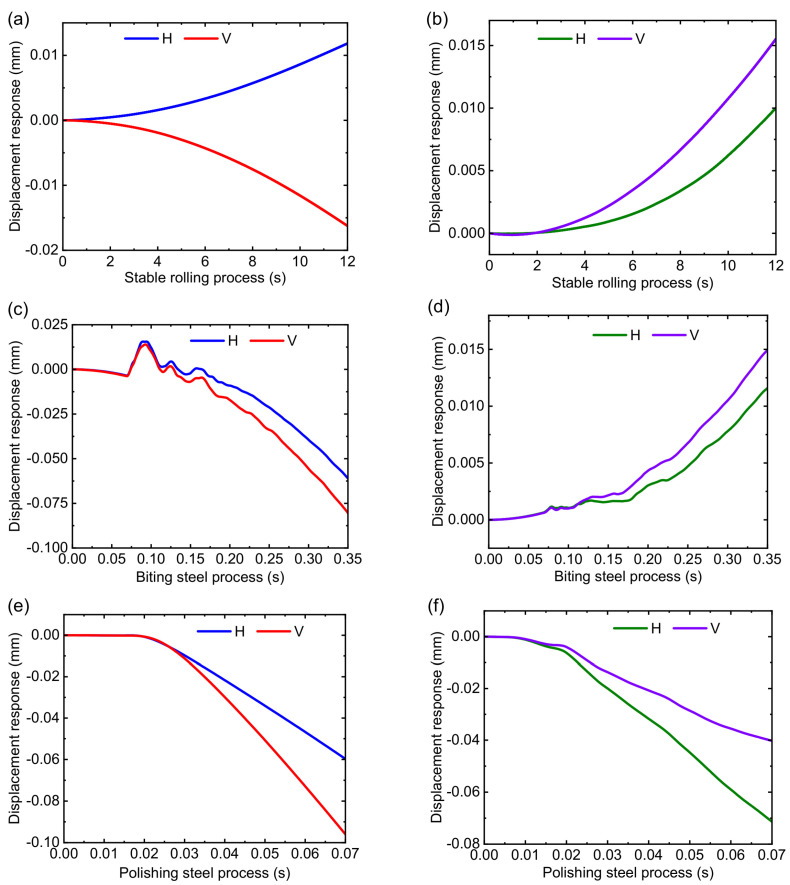
Transient displacement response of the system when the finished weathering steel thickness is 1.6 and 3.2 mm: (**a**) stable rolling process of the 1.6 mm, (**b**) stable rolling process of the 3.2 mm, (**c**) biting steel process of the 1.6 mm, (**d**) biting steel process of the 3.2 mm, (**e**) polishing steel process of the 1.6 mm, and (**f**) polishing steel process of the 3.2 mm.

The displacement response of 1.6 and 3.2 mm is shown in Figure 15. Regardless of whether the thickness of the finished product is 1.6 or 3.2 mm, the horizontal displacement response and vertical displacement response fluctuate within the range of 0.02 mm, which is mainly due to the fluctuation in the thickness of the strip or the relative motion between the components inside the support bearing. Instead of focusing on the specific value of the displacement, we only focus on the overall trend. As can be seen from the overall change trend, the direction of displacement fluctuation is different in the whole rolling process but the change in each direction is consistent with the numerical results. Once again, the numerical result is a coupled motion result of the system in all directions, whereas the experimental result is the result of each test direction.

## 5. Conclusions

For the first time, we established a nonlinear dynamic model of the RBBH system supported by four-row rolling bearings to better understand the dynamic characteristics of the system. In order to verify the accuracy of the dynamic model, we innovatively designed and assembled an engineering test platform on the production site to collect the vibration data of the system. Finally, the dynamic characteristics of the RBBH system are studied by the method of dynamic model and vibration data fusion. The following conclusions are obtained:

(1) The dynamic characteristics of the RBBH system are controlled by the dynamic model during the rolling period, including acceleration response, velocity response, and displacement response.
(2) A coupling nonlinear dynamic model and a general dynamic equation of the RBBH system supported by four-row rolling element bearings are proposed under high speed and heavy load.
(3) When the SPA-H of 1.6 mm is rolled, the dynamic response of the RBBH system is much greater than that of the 3.2 mm. The stability of the RBBH system varies with strip thickness.
(4) The dynamic characteristic of the RBBH system is a coupling dynamic result which contains the motion characteristic of multiple directions.

## Figures and Tables

**Table 1:** Structural parameters of the RBBH system.

Parameters	Values
Bearing width *B* (mm)	298.4
Inner ring diameter *D*_i_ (mm)	288.9
Outer ring diameter *D*_o_ (mm)	406.4
Rolling element small diameter *D*_r1_ (mm)	26.2
Rolling element big diameter*D*_r2_ (mm)	27.9
rolling element length *l*_r_ (mm)	54
Load ratings *C*_r_ (KN)	2627
Radial clearance *P*_d1_ (mm)	0.12–0.18
Axial clearance *P*_d2_ (mm)	0.54–0.79
Total length of roll *L* (mm)	5250
Barrel length *L*_b_ (mm)	2080
Barrel diameter *D*_b_ (mm)	760

**Table 2:** Material parameters of the RBBH system.

Component	Material Properties	Density (kg/m^3^)	Elastic Modulus (GPa)	Poisson’s Ratio
Spacer	SAE1045	7850	210	0.31
Cage	08Al	7800	210	0.3
Rolling element	G20Cr2Ni4	7810	209	0.32
Inner ring	G20Cr2Ni4	7810	209	0.32
Outer ring	G20Cr2Ni4	7810	209	0.32
Bearing housing	ZG230	7800	202	0.3
Roll	60CrMnMo	7870	207	0.25

## Data Availability

The experimental data can be obtained on request from (Shuilin Lin) lslin@stumail.ysu.edu.cn. The experimental data is obtained through the rolling experiment of National Cold Rolling Strip Equipment and Process Engineering Technology Research Center of Yanshan University. The experimental results are reproducible. Relevant scholars can use similar experimental models or go to the National Cold Rolling Strip Equipment and Process Engineering Technology Research Center of Yanshan University to further verify the reliability of the experimental data.
